# Osteoporosis in adult patients with atopic dermatitis: A nationwide population-based study

**DOI:** 10.1371/journal.pone.0171667

**Published:** 2017-02-16

**Authors:** Ching-Ying Wu, Ying-Yi Lu, Chun-Ching Lu, Yu-Feng Su, Tai-Hsin Tsai, Chieh-Hsin Wu

**Affiliations:** 1 Department of Dermatology, Kaohsiung Municipal Ta-Tung Hospital, Kaohsiung Medical University Hospital, Kaohsiung, Taiwan; 2 Graduate Institute of Medicine, College of Medicine, Kaohsiung Medical University, Kaohsiung, Taiwan; 3 Department of Dermatology, Kaohsiung Veterans General Hospital, Kaohsiung, Taiwan; 4 Cosmetic applications and management department, Yuh-Ing Junior College of Health Care & Management, Kaohsiung, Taiwan; 5 Department of Orthopedics, Taipei Veterans General Hospital, Taipei, Taiwan; 6 Division of Neurosurgery, Department of Surgery, Kaohsiung Medical University Hospital, Kaohsiung Medical University, Kaohsiung, Taiwan; 7 Department of Surgery, Faculty of Medicine, College of Medicine, Kaohsiung Medical University, Kaohsiung, Taiwan; Garvan Institute of Medical Research, AUSTRALIA

## Abstract

The aim of this study was to investigate osteoporosis risk in atopic dermatitis (AD) patients. This study included patients in the Taiwan National Health Insurance Research dataset. The population-based study included all patients aged 20–49 years who had been diagnosed with AD during 1996–2010. In total, 35,229 age and gender-matched patients without AD in a 1:1 ratio were randomly selected as the non-AD group. Cox proportional-hazards regression and Kaplan–Meier analyses were used to measure the hazard ratios and the cumulative incidences of osteoporosis, respectively. During the follow-up period, 360(1.02%) AD patients and 127(0.36%) non-AD patients developed osteoporosis. The overall incidence of osteoporosis was4.72-fold greater in the AD patients compared to the non-AD patients (1.82 vs. 0.24 per 1,000 person-years, respectively) after adjusting for potential confounding factors. Osteoporosis risk factors included female gender, age, advanced Charlson Comorbidity Index, depression and use of corticosteroids. The dataset analysis showed that AD was significantly associated with subsequent risk of osteoporosis.

## Introduction

Atopic dermatitis (AD), a chronic inflammatory skin disease associated with high morbidity, can substantially affect the quality of life of affected individuals and their families.[1] This disease is the most common chronic inflammatory skin disease.[2] Significant geographic variation in the prevalence of AD has been reported in both adults (2%–10%) and children (15%–30%).[3,4] Most of AD patients first develop the disease in childhood, adult- onset AD has been recognized as well.[5] Notably, the incidence of AD has increased in recent decades, especially in industrialized nations.

Osteoporosis is a systemic metabolic bone disease that has multiple causes and is characterized by a progressive loss of bone mass and microarchitecture resulting in a high fracture risk.[6] Previous studies have shown that bone mineral density (BMD) may be related to the incidence of chronic inflammatory diseases such as rheumatoid arthritis (RA),inflammatory bowel disease (IBD), systemic lupus erythematosus (SLE), psoriasis and AD.[7–14] In a recent study of 3141 IBD patients and 12,564 age- and gender-matched controls enrolled from 2000 to 2010, M.-S. Tsai et al. reported that the IBD group had a higher incidence of osteoporosis (adjusted hazard ratio [HR], 1.32; 95% confidence interval [CI], 1.09–1.60).[9] In Keller et al., the association between psoriasis and subsequent osteoporosis risk was investigated in a nationwide population of 17,507 osteoporosis patients. In comparison with 52,521 age- and gender-matched controls, the osteoporosis patients had an adjusted odds ratio of 1.65 for prior psoriasis. [10] Previous studies also indicate that loss of bone density culminating in fracture and osteoporosis is a common comorbidity in SLE. Osteoporosis occurs in 1.4–68% of SLE patients.[11,12] Haugeberg et al. found that the prevalence of osteoporosis is approximately 2-fold higher in subjects with RA compared to the general population.[13,14] In people with chronic inflammatory diseases, many proinflammatory cytokines, including Interleukin (IL)-6, IL-17,tumor necrosis factor (TNF) and interferon (IFN), are of important roles in the pathogenesis of osteoporosis.[10,15] Nevertheless, data on the risk of osteoporosis in patients with AD are scarce. In a study of 125 adult AD patients, I.M. Haeck et al. reported a high prevalence of low BMD in approximately one third of patients with moderate–to-severe AD. These patients were relatively young and predominantly male.[16] As increased blood levels ofIL-6, IL-8, IL-17,IL-18, TNF and IFN were also found in AD patients, we hypothesized that AD people are vulnerable to osteoporosis.[4,17] Therefore, this study used the Taiwan National Health Insurance (NHI) database to determine whether AD is a risk factor for osteoporosis.

## Patients and methods

### Database

The Taiwan NHI program is a mandatory single payer system implemented on March 1,1995. According to the Bureau of National Health Insurance (BNHI), approximately 99% of the 23.74 million residents of Taiwan are covered by the NHI program. The BNHI has authorized the National Health Research Institutes to create the National Health Insurance Research Database (NHIRD) and an encrypted secondary database for medical research; this database contains administrative and health claims data collected through the NHI program, including complete information on inpatient care, ambulatory care, gender, date of birth and prescriptions dispensed at contracted pharmacies. Researchers can access relevant claims information, but patient identification numbers are scrambled. This study used the Longitudinal Health Insurance Database, 2010 (LHID2010), which is a subset of the NHIRD comprising patient data from 1996 to 2010. The LHID2010 comprises data for 1,000,000 beneficiaries randomly sampled from the original NHIRD. This database enabled an analysis of osteoporosis risk in a large sample of AD patients. Osteoporosis and AD were defined according to the criteria specified in the International Classification of Diseases, Ninth Revision, Clinical Modification (ICD-9-CM).

### Ethical approval

This study analyzed insurance reimbursement claims data contained in the NHIRD, which is available for research purposes. The study was performed in accordance with Declaration of Helsinki guidelines. The study was also evaluated and approved by the Institutional Review Board of Kaohsiung Medical University Hospital (KMUHIRB-EXEMPT (I)-20150040).

### Study population

The study cohort included 35,229 patients aged 20–49 years [18] who had been diagnosed with AD (ICD-9-CM code 691) [19,20] during 1996–2010. For data accuracy, the cohort was limited to patients who had received at least two AD diagnoses during ambulatory visits [single-visit (n = 195) excluded] or patients who had received at least one AD diagnosis during an inpatient visit. Additionally, the cohort was limited to patients with ICD-9codes assigned by a dermatologist. The index date was designated as the date of the first clinical visit for AD. To ensure the accuracy of the data, we only included cases if they received ≥2 osteoporosis diagnoses for ambulatory visits [single-visit (n = 39) excluded]or ≥1 diagnosis in inpatient care and the ICD-9 code was assigned by orthopedists and receiving at least one BMD examination were included in the osteoporotic group.[10,21,22] Exclusion criteria were diagnosis of osteoporosis (ICD-9-CM code 733)[23] before or on the index date, incomplete data, and age younger than 20 years or older than 50 years. Based on the event rate, this study has more than 99% power (α = 0.05) to detect a significant association between AD and the later developmentof osteoporosis.The patients in the non-AD cohort were selected by a simple random sampling method. One insured person was randomly selected from the NHI beneficiaries without AD and frequency matched with every person with AD in the same period according to age, gender and index year, which was the year of AD diagnosis.

### Outcome and comorbidities

The patients in both the AD and non-AD cohorts were followed up until one of the following events occurred: diagnosis with osteoporosis; censor after loss to follow up, withdrawal of insurance coverage. Otherwise, all patients were followed up until the end of 2010. The potential confounding factors in this study were mainly determined according to Japanese 2011 guidelines for prevention and treatment of osteoporosis.[16,23–26] The following baseline comorbidities were identified according to diagnoses in the claims records data before the index date: hypertension (ICD-9-CM codes 401–405), diabetes mellitus (ICD-9-CM code 250), hyperlipidaemia (ICD-9-CM code 272), chronic kidney disease (ICD-9-CM code 582,583,585,586 and 588), chronic liver disease (ICD-9-CM code 456, 571 and 572), chronic obstructive pulmonary disease (ICD-9-CM code 491, 492, 494 and 496), depression (ICD-9-CM code 296.2, 296.3, 300.4 and 311). The Charlson comorbidity index (CCI) scores, categorised into four levels (0, 1–2, 3–4 and ≥5), were used to assess the severity of co-morbidities, which included myocardial infarction, congestive heart failure, peripheral vascular disease, cerebrovascular disease, dementia, chronic pulmonary disease, rheumatic disease, peptic ulcer disease, mild and moderate or severe liver disease, diabetes with and without chronic complication, hemiplegia or paraplegia, renal disease, any malignancy (including lymphoma and leukemia, except malignancy of skin), metastatic solid tumor, human immunodeficiency virus infection and acquired immune deficiency syndrome. The analysis also included the use of corticosteroids, within 5 years before the index date. [16,26]

### Statistical analysis

A chi-square test was used to compare distributions of categorical demographics and clinical characteristics between the AD and non-AD cohorts. The Student *t* test and Wilcoxon rank-sum test were used to compare mean age and follow-up time (y) between the 2 cohorts, as appropriately. The Kaplan-Meier method was used to estimate cumulative incidence, and the differences between the curves were tested by 2-tailed log-rank test. Survival was calculated until the occurrence of hospitalization, an ambulatory visit for osteoporosis, or the end of the study period (December 31, 2010), whichever came first. Incidence rates of osteoporosis were estimated in 1000 person-years and compared in both cohorts. Univariable and multivariable Cox proportional hazard regression models were used to investigate the HR and 95% CI for osteoporosis if the proportional hazards assumption was satisfied. The multivariable Cox models were adjusted for age, CCI, and relevant comorbidities. A 2-tailed P-value of <0.05 was considered statistically significant. All data processing and statistical analyses were performed using Statistical Analysis Software, version 9.4 (SAS Institute, Cary, NC, USA).

## Results

### Baseline characteristics of patients with and without AD

**Fig 1:**
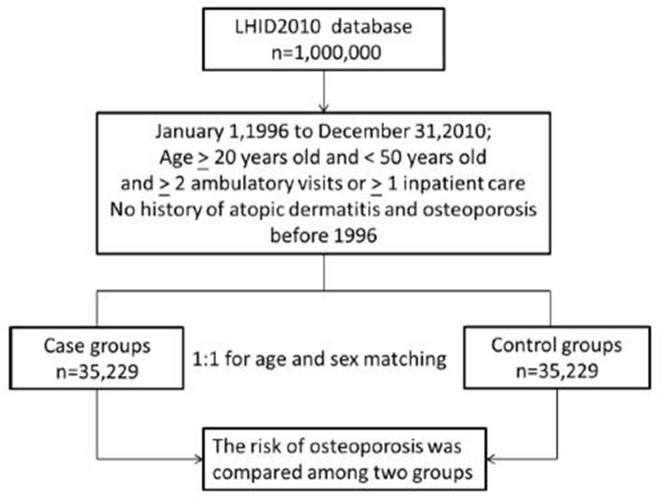
Flow diagram of the present study from the National Health Insurance Research Database in Taiwan. LHID = Longitudinal Health Insurance Database.

Fig 1 shows that the 70,458 patients enrolled between January, 1996 and December, 2010, including 35,229 people in the control (non-AD) group and 35,229 people in the case (AD) group.

Table 1 compares demographic characteristics and baseline comorbidities between the two cohorts. In the study cohort, 66.31% were female. The percentage of patients with each comorbidity was significantly higher in the AD group than in the non-AD group, including hypertension (15.10 vs. 6.56, p <0. 001), diabetes mellitus (9.65 vs. 4.17, P<0.001), hyperlipidaemia (20.76 vs.9.56, P<0. 001), chronic kidney disease (5.5.61vs. 2.78, P<0. 001), chronic liver disease (29.2vs. 16.71, P<0. 001), chronic obstructive pulmonary disease (14.87 vs. 8.33, P<0. 001),depression (11.46 vs. 6.35, P<0.001).The AD group had a relatively higher CCI. The use of corticosteroids was also significantly higher in the AD group compared to the non-AD group (24.61 vs.11.67, P<0. 001). Of the 35,229 AD patients, 360 (1.02%) had osteoporosis during a median observation time of 3.0 years (interquartile range [IQR] = 1.3–5.3).The incidence of osteoporosis was significantly (P<0. 001)higher in the AD group compared to 35,229 age- and gender-matched controls without AD (0.36%) during a median observation time of 8.3 years (IQR = 5.3–11.9). The development of osteoporosis was also significantly faster in the AD group (3.0 years) compared to the control group (8.3 years) during the following periods.

**Table 1:** Baseline characteristics of patients with and without atopic dermatitis in Taiwan.

Variables	Atopic dermatitis		P value
	Yes	No	
	N = 35,229	N = 35,229	
**Osteoporosis patients, n (%)**	360(1.02)	127(0.36)	<0.001
**Period of developing osteoporosis median (IQR**^**a**^**), years**	3.0(1.3–5.3)	8.3(5.3–11.9)	<0.001
**Age mean (SD**^**b**^**), years**	33.6 (8.4)	33.7 (8.4)	0.739
**Age group, n (%)**			
20–29	13929(39.54)	13929(39.54)	
30–39	11827(33.57)	11827(33.57)	
40–49	9473(26.89)	9473(26.89)	1.000
**Gender, n (%)**			
Men	11867(33.69)	11867(33.69)	
Women	23362(66.31)	23362(66.31)	1.000
**Charlson Comorbidity Index, n (%)**			
0	10118(28.72)	17145(48.64)	
1–2	17152(48.69)	14614(41.48)	
3–4	5795(16.45)	2808(7.97)	
≥5	2164(6.14)	662(1.88)	<0.001
**Co-morbidity, n (%)**			
** Hypertension**	5319(15.10)	2311(6.56)	<0.001
** Diabetes mellitus**	3399(9.65)	1468(4.17)	<0.001
** Hyperlipidaemia**	7315(20.76)	3367(9.56)	<0.001
** Chronic kidney disease**	1975(5.61)	980(2.78)	<0.001
** Chronic liver disease**	10290(29.21)	5886(16.71)	<0.001
** Chronic obstructive pulmonary disease**	5238(14.87)	2934(8.33)	<0.001
** Depression**	4038(11.46)	2238(6.35)	<0.001
**Medication, n (%)**			
** Systemic corticosteroids** ^**c**^	8671(24.61)	4111(11.67)	<0.001

^a^ IQR: interquartile range

^b^ SD: standard deviation

^c^ prednisolone equivalent to 5 mg daily more than 1 week.

### Osteoporosis incidence and risk

Table 2 compares the incidence and HRs of osteoporosis by gender, age, and comorbidity. During the follow-up period, osteoporosis was diagnosed in 360 (1.02%) AD patients and in 127 non-AD (0.36%) patients. The overall incidence of osteoporosis was 4.72 times greater in the AD group than in the non-AD group (1.82 vs. 0.24 per 1,000 person-years, respectively) after adjusting for age, CCI, related comorbidities (hypertension, diabetes mellitus, hyperlipidaemia, chronic kidney disease, chronic liver disease, chronic obstructive pulmonary disease, depression, and use of systemic corticosteroids. Gender-specific analyses showed that, in both cohorts, the incidence of osteoporosis was higher in women than in men in both the AD cohort (2.39 per 1,000 person-years in women vs. 0.78 in men) and in the non-AD cohort (0.29 per 1,000 person-years in women vs.0.15 in men). In addition, the osteoporosis risk was significant in both women and men (adjusted HR = 4.68 vs.4.73, respectively, P<0.001)

**Table 2:** Incidence and hazard ratios of osteoporosis by demographic characteristics and comorbidity among patients with or without atopic dermatitis.

Variables	Patients with atopic dermatitis			Patients without atopic dermatitis			Compared to Non-Atopic dermatitis	
	Osteoporosis	PY ^a^	Rate(95% CI)^b^	Osteoporosis	PY ^a^	Rate(95% CI)^b^	Crude HR(95% CI) ^c^	Adjusted HR(95% CI) ^c^
**All**	360	197301.06	1.82(1.82–1.83)	127	527603.39	0.24(0.24–0.24)	8.01 (6.32–10.16)^d^	4.72 (3.68–6.05)^d^
**Gender**								
Men	54	69316.34	0.78(0.77–0.78)	27	177872.66	0.15(0.15–0.15)	7.65 (4.22–13.85)^d^	4.68 (2.52–8.72)^d^
Women	306	127984.71	2.39(2.38–2.40)	100	349730.73	0.29(0.28–0.29)	8.23 (6.35–10.66)^d^	4.73 (3.61–6.20)^d^
**Stratify age**								
20–29	17	79086.29	0.21(0.21–0.22)	7	208887.09	0.03(0.03–0.03)	6.47(2.78–16.45)^d^	4.94 (1.87–11.13)^d^
30–39	63	65015.92	0.97(0.96–0.98)	28	177239.20	0.16(0.15–0.16)	6.76 (4.08–10.27)^d^	4.57 (2.53–6.52)^d^
40–49	280	53198.84	5.26(5.22–5.31)	92	223284.74	0.65(0.65–0.65)	8.52 (6.53–11.12)^d^	4.03(3.15–6.43)^d^
**Comorbidity**								
No	20	45399.59	0.44(0.44–0.45)	18	230253.72	0.08(0.07–0.08)	5.93 (3.09–11.34)^d^	5.61 (2.93–10.78)^d^
Yes	340	151901.47	2.24(2.23–2.25)	109	297349.68	0.37(0.36–0.37)	6.42 (5.01–8.23)^d^	4.56 (3.52–5.89)^d^

^a^ PY, person-years.

^b^ Rate, incidence rate in per 1000 person-years.

^c^ HR, hazard ratio; 95% CI, 95% confidence interval; adjusted HR, adjusted for age, sex, comorbidities (hypertension, diabetes mellitus, hyperlipidaemia, chronic kidney disease, chronic liver disease, chronic obstructive pulmonary disease, depression) and use of systemic corticosteroids.

^d^ P<0.001.

The incidence of osteoporosis was consistently higher in the AD group at different ages, and the incidence rate consistently increased with age. Compared with the non-AD group, the AD group had a higher osteoporosis risk in all age groups. Regardless of comorbidities, the patients with AD exhibited a higher risk of osteoporosis than that of the non-AD patients.

**Fig 2:**
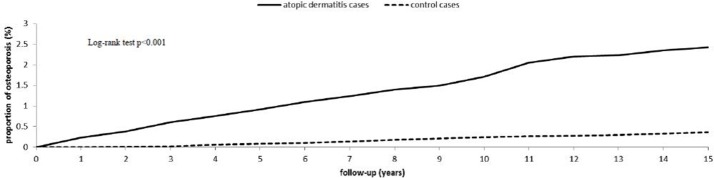
Cumulative incidence of osteoporosis for adult patients with atopic dermatitis and the general population control cohort.

Fig 2 shows the Kaplan-Meier curve for the cumulative incidence of osteoporosis in patients with and without AD after 15 years of follow up. The cumulative incidence of osteoporosis for the two cohorts showed that the AD incidence curve is significant higher than that for the control cohort (log rank test P <0.001.).

The Cox regression analysis results are shown in Table 3, which revealed female gender, age, advanced Charlson Comorbidity Index, depression, use of systemic corticosteroids are independent predictors of osteoporosis in AD patients)

**Table 3:** Risk factor analysis of osteoporosis in patients with atopic dermatitis.

Variables	Adjusted HR ^a^	(95% CI)^b^	P-value
Age (years)	2.02	(1.84–2.20)	<0.001
Female gender	3.91	(2.92–5.23)	<0.001
Charlson Comorbidity Index	1.73	(1.52–1.95)	<0.001
Depression	1.50	(1.18–1.89)	<0.001
Systemic corticosteroids use	1.42	(1.14–1.76)	0.001

^a^HR, hazard ratio; adjusted HR, adjusted for age, sex, comorbidities (hypertension, diabetes mellitus, hyperlipidaemia, chronic kidney disease, chronic liver disease, chronic obstructive pulmonary disease, depression) and use of systemic corticosteroids.

^b^ 95% CI,95% confidence interval.

The adjusted HR and 95% CI were estimated by a stepwise the Cox proportional hazards regression method.

## Discussion

The link between AD and osteoporosis has not been well studied. To the best of our knowledge, this study is the first to investigate the relationship between AD and subsequent osteoporosis in a nationwide population in Asia. During the follow-up period, 360 patients with AD (1.02%) and 127 non-AD participants (0.36%) developed osteoporosis. After adjusting for potential confounding factors, the overall incidence of osteoporosis was 4.72-times greater in the AD group than in the control group (1.82 vs. 0.24 per 1,000 person-years, respectively). This population-based cohort study of AD patients revealed that osteoporosis risk was significantly associated with female gender, older age, a high CCI score, depression and use of systemic corticosteroids.

A conflicting association between AD and low BMD has been reported in the previous studies.[16,26–29] For example, a study of 28 adults with AD by Aalto- Korte et al. found that, in11% of the AD patients, the Z-score for low BMD was less than –2. They concluded that BMD did not significantly differ between AD patients and healthy controls.[27] On the other hand, a study of 125 adult patients with moderate-to-severe AD by I.M. Haeck et al. reported that approximately one third of the patients had low BMD. The authors reported that 32.8% had osteopenia, and 4.8% had osteoporosis. Notably, the cumulative dose of oral corticosteroids taken within 5 years prior to the study did not significantly differ between patients with a low BMD and patients with normal BMD.[16]

The mechanisms that predispose AD patients to osteoporosis are multifactorial. A growing body of evidence indicates that AD is associated with depression. In a comparison of mood symptoms between 45 AD patients and 34 healthy controls, Hashiro and Okumura showed that the AD patients had a higher prevalence of depression symptoms and psychosomatic symptoms.[30] In an analysis of data for 8208 AD patients contained in the Taiwan NHIRD, C.-M. Cheng et al. showed that AD patients have an elevated risk of and depressive disorder (HR:5.44, 95%CI:3.99–7.44), anxiety disorders (HR:3.57,95%CI:2.55–4.98), and major depression (HR: 6.56,95%CI:3.64–11.84). Both adolescents and adults with AD have an increased risk of major depression, any depressive disorder, and anxiety disorders in later life.[31]A meta-analysis of six case controlled and eight cross-sectional studies (N = 10,523)by Q. Wu and colleagues revealed that depression is associated with low BMD.[32]Therefore, we concluded that AD patients are associated with an increased risk of osteoporosis through the possible strong correlation of depression. Second, studies of the various etiological factors responsible for the increased prevalence of atopic diseases in recent decades have revealed the important role of vitamin D. Although the literature is inconsistent, several recent reports indicate that vitamin D plays a role in the pathogenesis of AD. In a case-control study of obese patients, Oren et al compared 100 and 190 patients with and without vitamin D deficiency, respectively, out of 717 patients attending a weight loss center. Atopic dermatitis was diagnosed in 5% of patients with vitamin D deficiency but in only 1% of patients without vitamin D deficiency.[33] Another review by Krishna Mutgi and John Koo identified an inverse relationship between vitamin D levels and the severity of AD. A vitamin D deficiency can damage the integrity of the epidermal barrier.[34]Vitamin D also plays a major role in bone health in all age groups. Low vitamin D levels can substantially reduce bone mass, which then leads to osteoporosis.[35] A third cause of bone loss in AD patients is the inflammatory nature of the disorder, especially in patients with inadequate control of disease activity. Atopic dermatitis may be analogous to other chronic inflammatory diseases such as IBD and RA, in which the inflammatory process is known to be an independent risk factor for bone loss.[9,36] Recently, Silverberg used data from the 2005–2006 National Health and Nutrition Examination Survey (n = 4970) and from the National Health Interview Survey in 2010 (n = 27157) and in 2012 (n = 34525) to determine whether adult AD is associated with cardiovascular disease (CVD) risk. The report concluded that adults with AD may have significantly higher than normal odds of cardiovascular disease, heart attack, and congestive heart failure.[37] Previous studies in Taiwan and Sweden have reported that CVD is associated with risk of major osteoporotic fracture[38–40] which implies that AD is associated with osteoporosis. AD is also associated with obesity[41,42], cigarette smoking[41], and high alcohol consumption[41] all of which considered risk factors for osteoporosis.[43,44] These behavioral factors may at least partially explain the relationship between AD and osteoporosis.

The strength of this cohort study is the use of a population-based dataset comprising a large number of patients. Although the data suggest that AD is associated with an increased risk of osteoporosis, several limitations must be considered when interpreting these findings. First, the NHIRD does not provide detailed patient data for critical confounding variables such as habitual consumption of tobacco or alcohol, body mass index, physical activity, and family history. Second, the main purpose of the diagnostic codes in the NHI claims data is administrative billing. Medical records and diagnoses of AD and osteoporosis could not be reviewed and verified by ourselves. Validating the diagnoses of osteoporosis was difficult because we could not evaluate osteoporosis directly through densitometry. However, diagnostic accuracy was enhanced by limiting the study population to patients who had received medical care for osteoporosis on ≥2 separate visits and at least one BMD examination. The diagnoses of osteoporosis were made by physicians according to standard clinical criteria and were recorded using ICD-9 codes. Furthermore, medical experts of the Professional Peer Review Committee at the BNHI conduct regular scrutinization to ascertain the accuracy of diagnostic codes used in health insurance claims in Taiwan. Doctors and hospitals are motivated to enter diagnostic codes accurately because they are subject to big fines for incorrect entries. Finally, the diagnoses and codes for osteoporosis used in our study should be at least as reliable as those used in previous studies.[21,23,45] Third, patients with AD in Taiwan may not visit a specialist but may seek alternative medicine or receive treatment privately, possibly underestimating the real incidence of AD. Finally, the quality of a statistical analysis of data derived from a retrospective cohort study is generally lower than that of data obtained in randomized trials because of potential biases related to adjustments for confounding variables.

In summary, this study revealed an increased risk of osteoporosis in AD patients, and AD might be an early predictor of osteoporosis. Since measuring BMD is an easily performed and noninvasive way to evaluate osteoporosis, alerting physicians to this association may improve early identification of osteoporosis in patients with AD.
